# Comparison of flap and contralateral donor site tissue perfusion for flap monitoring in microvascular head and neck reconstruction – a retrospective study

**DOI:** 10.1016/j.jpra.2025.12.020

**Published:** 2025-12-17

**Authors:** Mark Ooms, Philipp Winnand, Marius Heitzer, Nils Vohl, Anna Bock, Marie Sophie Katz, Frank Hölzle, Ali Modabber

**Affiliations:** Department of Oral and Maxillofacial Surgery, University Hospital RWTH Aachen, Pauwelsstraße 30, 52074 Aachen, Germany

**Keywords:** Microvascular free flap, Head and neck reconstruction, Flap monitoring, Flap perfusion, Donor site

## Abstract

**Introduction:**

Postoperative flap monitoring in microvascular head and neck reconstruction based solely on flap tissue perfusion measurements relative to predefined thresholds may be influenced by systemic tissue perfusion alterations. This study compared flap and contralateral donor site tissue perfusion and evaluated the utility of the flap-to-donor site ratio in detecting vascular flap compromise.

**Materials & methods:**

Tissue perfusion measured with the O2C analysis system at a tissue depth of 3 mm at 0, 12, 24, 36, and 48 h postoperatively was retrospectively analyzed in 62 patients who underwent head and neck reconstruction with a fasciocutaneous free flap (FFF) (radial free forearm flap) or a perforator free flap (PFF) (anterolateral thigh flap or fibula free flap) between 2020 and 2022. Flap and contralateral donor site tissue perfusion parameters (blood flow, hemoglobin concentration, and hemoglobin oxygen saturation) were compared, and flap-to-donor site ratio cut-off values indicating vascular flap compromise, based on the minimum (blood flow and hemoglobin oxygen saturation) or maximum (hemoglobin concentration) value of all 5 measurement timepoints, were determined.

**Results:**

Blood flow, hemoglobin concentration, and hemoglobin oxygen saturation were in general higher at the flap than the donor site (*p* < 0.05). Cut-off values for the flap-to-donor site ratio indicating vascular flap compromise were as follows for FFFs and PFFs, respectively: <1.063 and <0.224 for blood flow, >1.792 and >1.597 for hemoglobin concentration, and <0.673 and <0.199 for hemoglobin oxygen saturation (sensitivity all 100 % [confidence interval (CI) range 2.5–100.0 %]; specificity all >65 % [CI range 49.0–100.0 %]).

**Conclusion:**

The postoperative course of tissue perfusion differs between flap and contralateral donor sites in FFFs and PFFs. The flap-to-donor site ratio could be used to detect vascular flap compromise in microvascular head and neck reconstruction, with further studies needed to confirm the determined cut-off values for the flap-to-donor site ratio indicating vascular flap compromise.

## Introduction

Microvascular free flap reconstruction facilitates aesthetic and functional tissue restoration with high success rates and is an established method for reconstruction of the head and neck region.^1^^,^^2^ However, free flap failure due to vascular flap compromise still occurs.^2^^,^^3^

Microvascular flap perfusion is a prerequisite for flap viability and is initially solely dependent on patent microsurgical anastomosis, with vascular flap compromise leading to insufficient flap perfusion and, consequently, flap failure.^4^, ^5^, ^6^ As the time interval between the occurrence and correction of vascular flap compromise to regain sufficient perfusion is inversely related to flap salvage, postoperative flap monitoring is mandatory.^7^, ^8^, ^9^ In this context, technical methods for postoperative flap monitoring were developed, as clinical flap monitoring as the gold standard, based on the assessment of flap color, temperature, capillary refill, and turgor, is associated with disadvantages, such as the need for clinical experience and the time delay between the onset and clinical signs of vascular flap compromise.^5^^,^^7^^,^^10^, ^11^, ^12^

The O2C analysis system (O2C Oxygen-to-see, LEA Medizintechnik, Giesen, Germany), which uses Doppler spectroscopy (830 nm; 30 mW) to determine blood flow (AU) and white light spectroscopy (500–800 nm; 50 W) to determine hemoglobin concentration (AU) and hemoglobin oxygen saturation (%), is a technical method for postoperative flap monitoring that uses surface probes to measure flap tissue perfusion and detect vascular flap compromise based on predefined thresholds for blood flow, hemoglobin concentration, and hemoglobin oxygen saturation.^7^^,^^13^^,^^14^ However, frequent postoperative alterations in blood pressure and vessel diameter, both of which are determinants of systemic tissue perfusion, likely influence flap tissue perfusion independent of microsurgical anastomosis and thus might limit the accuracy of flap monitoring.^7^^,^^14^, ^15^, ^16^, ^17^, ^18^, ^19^, ^20^, ^21^

Therefore, measuring and comparing tissue perfusion between the flap (dependent on a patent microsurgical anastomosis) and the contralateral donor site as the reference (not dependent on a patent microsurgical anastomosis) in terms of flap-to-donor-site ratios may eliminate or at least attenuate these influences and increase the accuracy of postoperative flap monitoring, thereby possibly enabling earlier detection of vascular flap compromise.^5^^,^^7^^,^^11^^,^^14^^,^^15^ In this context, the use of flap-to-donor-site ratios contrasts to other methods of postoperative flap monitoring, such as for example serial clinical assessment, near-infrared spectroscopy, implantable Doppler device, or microdialysis, which only evaluate the flap site.^5^^,^^11^ In addition, the use of flap-to-donor site ratios differs from conventional postoperative flap monitoring with the O2C analysis system by providing absolute thresholds for all perfusion parameters, including hemoglobin concentration.^7^^,^^14^ Thus, using flap-to-donor-site ratios for postoperative flap monitoring with the O2C analysis system could improve its accuracy without substantially compromising its ease of use and cost-effectiveness.^7^^,^^14^

This study aimed to investigate the relationship between flap and contralateral donor site tissue perfusion and to evaluate the utility of the flap-to-donor site ratio of tissue perfusion in detecting vascular flap compromise.

## Materials and methods

### Materials

This study based on retrospectively analyzed data was performed in compliance with the World Medical Association Declaration of Helsinki (June 1964) and subsequent amendments and have been approved by the local ethics committee of the Medical Faculty of RWTH Aachen University (EK 22-358). In addition, the manuscript was checked against the Strengthening the Reporting of Observational Studies in Epidemiology (STROBE) checklist.

The study included 62 patients who underwent microvascular reconstruction in the head and neck region after resection of malignant or nonmalignant disease with either a fasciocutaneous free flap (FFF) (radial free forearm flap) or a perforator free flap (PFF) (anterolateral thigh flap or fibula free flap) in our Department of Oral and Maxillofacial Surgery between 2020 and 2022. The inclusion criteria were complete data sets and patients older than 18 years.

Baseline data were extracted from clinical records. Surgery duration was defined as the time between the first incision and the last suture, and flap ischemia duration was defined as the time between the interruption of flap perfusion at the donor site after dissection of the flap pedicle and the restoration of flap perfusion at the recipient site after anastomosis release. Flap revision was defined as positive in cases of surgical anastomosis revision with return to the operating room. The decision to revise a flap was independent of the data analyzed in this study and was based on routine flap monitoring with the O2C analysis system, with measurement of flap perfusion with an unattached surface probe at 2- and 8-mm tissue depths in relation to predefined thresholds (blood flow <20/<10 arbitrary units [AU] for FFFs and <15/<5 AU for PFFs; hemoglobin concentration increase of >30 % for FFFs and PFFs; hemoglobin oxygen saturation <15 % for FFFs and <10 % for PFFs.^7^^,^^14^ Flap survival was defined as negative in cases of flap removal due to tissue necrosis.

Surgical procedures were performed under general anesthesia. The patients were monitored in the intensive care unit with invasive mechanical ventilation and analgosedation postoperatively until at least the following morning.

### Methods

#### Tissue perfusion measurement data

**Figure 1:**
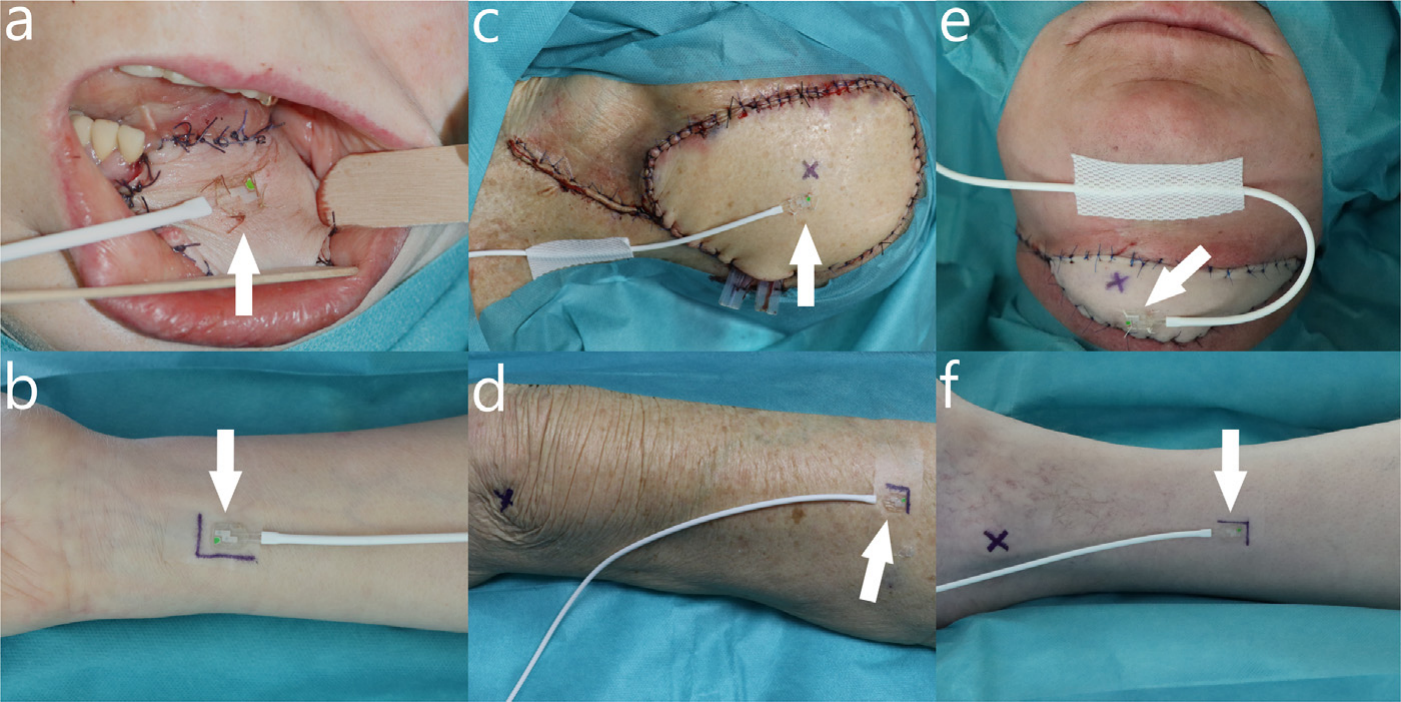
Measurement of tissue perfusion at the flap (a,c,e) and the contralateral donor site (b,d,f) for fasciocutaneous free flaps (radial free forearm flap [a,b]) and perforator free flaps (anterolateral thigh flap [c,d] and fibula free flap [e,f]) with the surface probe (white arrow) attached permanently at the flap with sutures and attached temporarily at the contralateral donor site with adhesive tape. Black crosses on perforator flaps represent the location of the perforator vessel (c,e) and the location of the superior lateral patella border (d) or the lateral malleolus (f) at the contralateral donor site. Measurement of tissue perfusion at the contralateral donor site was performed at a 4 cm distance from the hand palm (b) for radial free forearm flaps, at the center of an orientation line between the anterior superior iliac spine (not shown) and the superior lateral patella border (d) for anterolateral thigh flaps, or at the center between the middle and lower third of an orientation line between the lateral malleolus (f) and the fibula head (not shown) for fibula free flaps.

**Figure 2:**
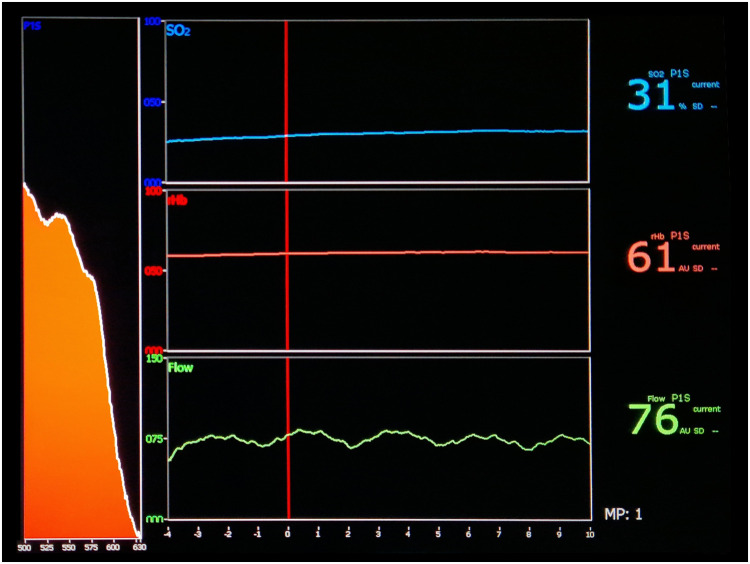
Representation of the measurement of tissue perfusion at the contralateral donor site of a fasciocutaneous free flap (radial free forearm flap) on the O2C analysis system (O2C Oxygen-to-see, LEA Medizintechnik, Giesen, Germany) display in terms of blood flow (bottom), hemoglobin concentration (middle), and hemoglobin oxygen saturation (top).

Tissue perfusion measurement data were obtained with the O2C analysis system. Tissue perfusion was measured with an attached surface probe at a 3-mm tissue depth for 10 s at 0, 12, 24, 36, and 48 h postoperatively at the flap and at the contralateral donor site (Figure 1, Figure 2). At the flap, a surface probe was attached permanently with four sutures in the flap center for FFFs and at a distance of 1 cm longitudinally parallel to the perforator vessel for PFFs. Patients with intraoral flaps used a bite wedge for at least 48 h postoperatively. At the contralateral donor site, a surface probe was attached temporarily to the skin with adhesive tape, and the surface probe was placed at the same location aligned with the permanent pen markings for each measurement. For FFFs, the location selected was at a distance of 4 cm from the palm and centered on the forearm, while for PFFs, the location selected was at the midpoint of an orientation line between the anterior superior iliac spine and the superior lateral patella border for anterolateral thigh flaps and halfway between the middle and lower third of an orientation line between the lateral malleolus and the fibula head for fibula free flaps.^22^, ^23^, ^24^

#### Statistical analysis

Baseline data were expressed as numbers (with percentage) or medians (with interquartile range), and tissue perfusion parameters (blood flow, hemoglobin concentration, and hemoglobin oxygen saturation) were expressed as median (with interquartile range). Flap-to-donor site ratios were calculated separately for each of the tissue perfusion parameters for each of the measurement timepoints (0, 12, 24, 36, and 48 h postoperatively) and each of the flap types (FFFs and PFFs). For non-revised flaps, the Wilcoxon signed rank test was used to test for differences in tissue perfusion parameters between the flap and contralateral donor sites. Additionally, the one sample Wilcoxon test was used to test for differences between the flap-to-donor site ratio of tissue perfusion of a revised flap and the flap-to-donor site ratio of tissue perfusion of all non-revised flaps of the same flap type. Receiver operator characteristics analyses were performed separately for each flap type and each tissue perfusion parameter with all measurement timepoints pooled (by taking the minimum [blood flow and hemoglobin oxygen saturation] or the maximum [hemoglobin concentration] value for all 5 measurement timepoints), and the theoretical optimal cut-off values for flap-to-donor site ratios of tissue perfusion to predict flap revision were determined by calculating the Youden index.^25^ Diagnostic accuracy was determined by calculating the area under the curve.^26^ Confidence intervals for the area under the curve were obtained by bootstrapping (*b* = 1000) and for test parameters such as sensitivity, specificity, positive predictive value and negative predictive value by the Clopper-Pearson method. *p*-values < 0.05 were considered statistically significant. Statistical analysis was performed using SPSS Version 28 (SPSS, IBM, New York, USA).

## Results

### Clinical characteristics of the study population

The study population included 62 patients, of which 39 were reconstructed with a FFF and 23 were reconstructed with a PFF (15 anterolateral thigh flaps and eight fibula free flaps) (Table 1). Flap revision was performed postoperatively in three FFFs due to venous congestion after 12 h in two cases and 36 h in one case and in one PFF due to arterial congestion after 12 h.

**Table 1:** Clinical characteristics of the study population.

*Number*	62
*Sex (n)*	
male	30 (48.4 %)
female	32 (51.6 %)
*Age (years)*	68.0 (17.0)
*BMI (kg/m²)*	24.7 (6.3)
*ASA (n)*	
1	0 (0.0 %)
2	22 (35.5 %)
3	38 (61.3 %)
4	2 (3.2 %)
*Flap type (n)*	
FFF	39 (62.9 %)
PFF	23 (37.1 %)
*Flap location (n)*	
intraoral	
tongue	8 (12.9 %)
mouth floor	6 (9.7 %)
mandibula	8 (12.9 %)
maxilla	4 (6.5 %)
cheek	3 (4.8 %)
extraoral	
submandibular	12 (19.3 %)
buccal	5 (8.1 %)
infraorbital	2 (3.2 %)
nasal	2 (3.2 %)
orbital	5 (8.1 %)
auricular	5 (8.1 %)
temporoparietal	2 (3.2 %)
*Arterial anastomosis recipient vessel (n)*	
external carotid artery	9 (14.5 %)
facial artery	40 (64.5 %)
lingual artery	1 (1.6 %)
superior thyroid artery	12 (19.4 %)
*Venous anastomosis recipient vessel (n)*	
internal jugular vein	36 (58.0 %)
internal jugular vein + other vein	13 (21.0 %)
other vein	13 (21.0 %)
*Surgery duration* (min)	512.0 (169.0)
*Flap ischemia duration* (min)	93.0 (30.0)
Flap revision (*n*)	
*no*	58 (93.5 %)
*yes*	4 (6.5 %)
*Flap survival (n)*	
*no*	1 (1.6 %)
*yes*	61 (98.4 %)

Parameters are indicated as numbers (with percentage) for categorical data (sex, ASA, flap type, flap location, arterial anastomosis recipient vessels, venous anastomosis recipient vessel, flap revision, flap survival) or median (with interquartile range) for metric data (age, BMI, surgery duration, flap ischemia duration); other vein = facial vein, superior thyroid vein, external jugular vein, retromandibular vein; abbreviations: BMI, Body mass index; ASA, American society of anesthesiologists score; FFF, Fasciocutaneous free flap; PFF, Perforator free flap.

### Comparison of tissue perfusion between the flap and the donor site in non-revised flaps

**Figure 3:**
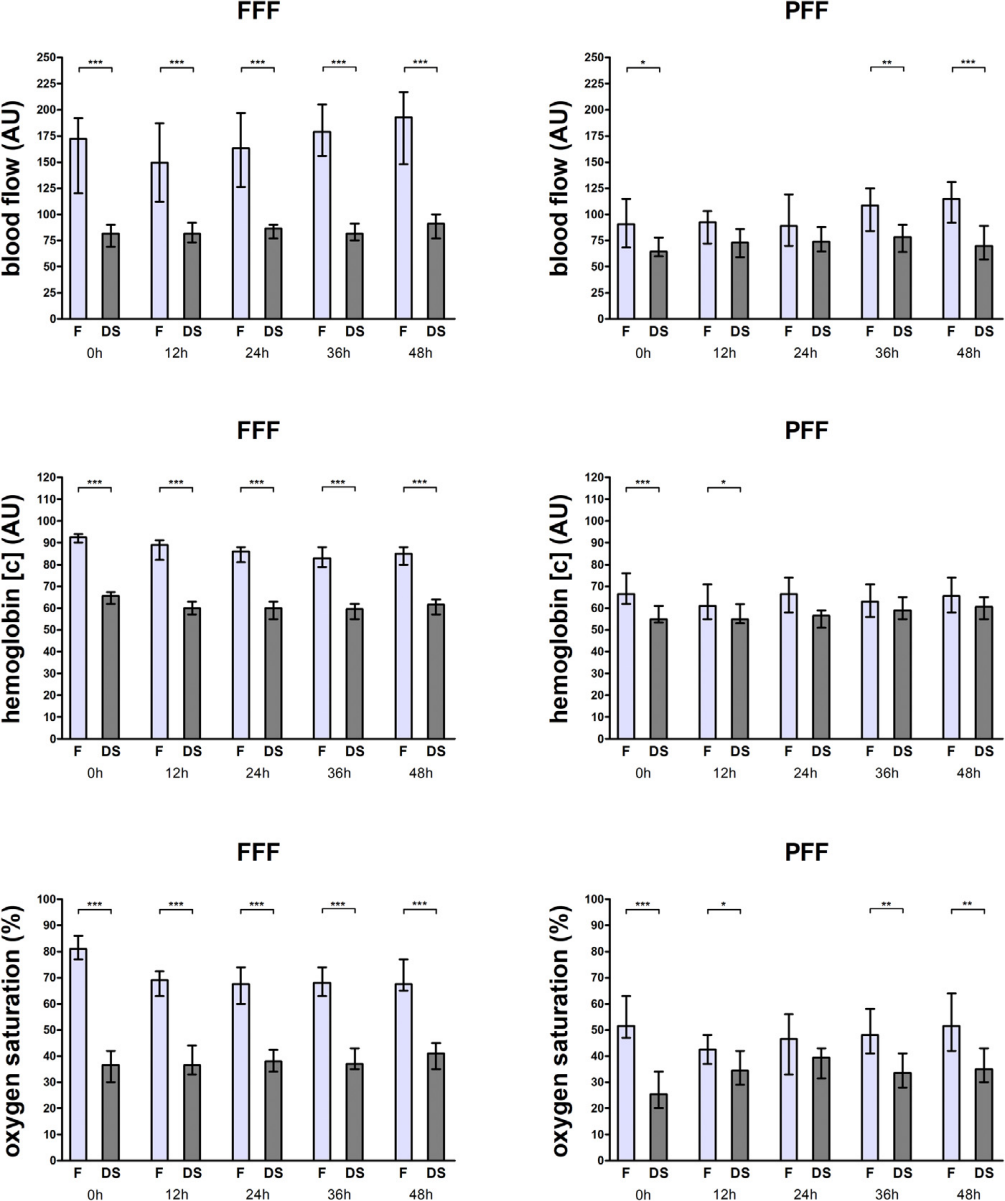
Data shown as median (with 95 % confidence interval) for blood flow (top), hemoglobin concentration (middle), and hemoglobin oxygen saturation (bottom) at the flap (F) and at the contralateral donor site (DS) for each measurement timepoint (0 h postoperatively, 12 h postoperatively, 24 h postoperatively, 36 h postoperatively, 48 h postoperatively) separately for non-revised fasciocutaneous free flaps (FFF) (left) and perforator free flaps (PFF) (right); *p*-values corresponding to testing for differences between tissue perfusion at the flap and tissue perfusion at the contralateral donor site with Wilcoxon signed rank test; ***p < 0.001, **p < 0.01, *p < 0.05; abbreviations: FFF, Fasciocutaneous free flap; PFF, Perforator free flap; AU, Arbitrary units, F, Flap, DS, Donor site.

Tissue perfusion differed between the flap and donor site in non-revised FFFs at all timepoints, with higher values at the flap for blood flow, hemoglobin concentration, and hemoglobin oxygen saturation (all *p* < 0.05) (Table 2, Figure 3). Tissue perfusion differed partially between the flap and donor site in non-revised PFFs, with higher values at the flap for blood flow at 0, 36, and 48 h, for hemoglobin concentration at 0 and 12 h, and for hemoglobin oxygen saturation at 0, 12, 36, and 48 h postoperatively (all *p* < 0.05) (Table 2, Figure 3).

**Table 2:** Tissue perfusion measurement values for non-revised flaps.

Timepoint	Flap	Donor site	Flap-to-donor site ratio	*p*-value
FFF (*n* = 36)				
Blood flow (AU)				
*0 h*	172.0 (131.0)	81.5 (31.0)	1.952 (1.60)	<0.001
*12 h*	149.5 (115.0)	81.5 (34.0)	1.971 (1.30)	<0.001
*24 h*	163.0 (90.0)	86.5 (27.0)	1.923 (1.40)	<0.001
*36 h*	179.0 (75.0)	81.5 (27.0)	1.982 (1.00)	<0.001
*48 h*	193.0 (90.0)	91.0 (31.0)	2.069 (1.10)	<0.001
Hemoglobin concentration (AU)				
*0 h*	92.5 (8.0)	65.5 (11.0)	1.389 (0.30)	<0.001
*12 h*	89.0 (14.0)	60.0 (12.0)	1.406 (0.30)	<0.001
*24 h*	86.0 (13.0)	60.0 (12.0)	1.363 (0.40)	<0.001
*36 h*	83.0 (14.0)	59.5 (12.0)	1.446 (0.30)	<0.001
*48 h*	85.0 (15.0)	61.5 (12.0)	1.406 (0.40)	<0.001
Hemoglobin oxygen saturation (%)				
*0 h*	81.0 (21.0)	36.5 (18.0)	2.150 (1.00)	<0.001
*12 h*	69.0 (17.0)	36.5 (19.0)	1.675 (1.10)	<0.001
*24 h*	67.5 (23.0)	38.0 (17.0)	1.600 (0.40)	<0.001
*36 h*	68.0 (16.0)	37.0 (13.0)	1.743 (0.60)	<0.001
*48 h*	67.5 (16.0)	41.0 (16.0)	1.713 (0.60)	<0.001
PFF (*n* = 22)				
Blood flow (AU)				
*0 h*	90.5 (65.0)	64.5 (29.0)	1.204 (1.40)	0.049
*12 h*	92.5 (36.0)	73.0 (35.0)	1.087 (1.10)	0.168
*24 h*	89.0 (62.0)	74.0 (43.0)	1.148 (0.90)	0.123
*36 h*	108.5 (55.0)	78.5 (32.0)	1.373 (0.70)	0.004
*48 h*	115.0 (55.0)	70.0 (33.0)	1.565 (1.00)	<0.001
Hemoglobin concentration (AU)				
*0 h*	66.5 (20.0)	55.0 (9.0)	1.165 (0.20)	<0.001
*12 h*	61.0 (18.0)	55.0 (11.0)	1.068 (0.40)	0.022
*24 h*	66.5 (17.0)	56.5 (15.0)	1.138 (0.40)	0.070
*36 h*	63.0 (16.0)	59.0 (13.0)	1.071 (0.40)	0.330
*48 h*	65.5 (18.0)	60.5 (15.0)	1.118 (0.40)	0.305
Hemoglobin oxygen saturation (%)				
*0 h*	51.5 (21.0)	25.5 (23.0)	1.965 (2.10)	<0.001
*12 h*	42.5 (17.0)	34.5 (16.0)	1.092 (0.90)	0.045
*24 h*	46.5 (25.0)	39.5 (20.0)	1.157 (1.20)	0.119
*36 h*	48.0 (21.0)	33.5 (18.0)	1.467 (1.10)	0.005
*48 h*	51.5 (24.0)	35.0 (24.0)	1.566 (1.10)	0.007

Data presented as median (with interquartile range) for tissue perfusion at the flap, for tissue perfusion at the contralateral donor site, and as flap-to-donor site ratio of tissue perfusion separately for each measurement timepoint (0 h postoperatively, 12 h postoperatively, 24 h postoperatively, 36 h postoperatively, 48 h postoperatively) for non-revised fasciocutaneous free flaps (FFF) and perforator free flaps (PFF); *p*-values corresponding to testing for differences between tissue perfusion at the flap and tissue perfusion at the contralateral donor site with Wilcoxon signed rank test; significant *p*-values are bold; abbreviations: AU, Arbitrary units; FFF, Fasciocutaneous free flap; PFF, Perforator free flap.

### Comparison of flap-to-donor site ratios of tissue perfusion between revised flaps and non-revised flaps

The flap-to-donor site ratios of tissue perfusion differed between revised and non-revised FFFs and PFFs at the measurement timepoint prior to flap revision, with lower values for blood flow and hemoglobin oxygen saturation and higher values for hemoglobin concentration in revised flaps (all *p* < 0.05) (Table 3).

**Table 3:** Tissue perfusion measurement values for revised flaps.

Timepoint	Flap	Donor site	Flap-to-donor site ratio	*p*-value
FFF (*n* = 3)				
Blood flow (AU)				
*12 h*	20	109	0.183	<0.001
*12 h*	94	75	1.253	<0.001
*36 h*	55	134	0.410	<0.001
Hemoglobin concentration (AU)				
*12 h*	106	59	1.797	<0.001
*12 h*	104	45	2.311	<0.001
*36 h*	98	59	1.661	<0.001
Hemoglobin oxygen saturation (%)				
*12 h*	28	47	0.596	<0.001
*12 h*	9	40	0.225	<0.001
*36 h*	6	30	0.200	<0.001
PFF (*n* = 1)				
Blood flow (AU)				
*12 h*	6	93	0.065	<0.001
Hemoglobin concentration (AU)				
*12 h*	85	61	1.393	<0.001
Hemoglobin oxygen saturation (%)				
*12 h*	1	29	0.034	<0.001

Data presented as absolute values for tissue perfusion at the flap, for tissue perfusion at the contralateral donor site, and as flap-to-donor site ratio of tissue perfusion separately for three revised fasciocutaneous free flaps (FFF) and one revised perforator free flap (PFF) corresponding to the measurement timepoint before flap revision (12 h postoperatively for three flaps and 36 h postoperatively for one flap); *p*-values corresponding to testing for differences between the flap-to-donor site ratio of tissue perfusion of the revised flap and the flap-to-donor site ratio of tissue perfusion of non-revised flaps of the same flap type with one sample Wilcoxon test; significant *p*-values are bold; abbreviations: AU, Arbitrary units; FFF, Fasciocutaneous free flap; PFF, Perforator free flap.

### Cut-off values for flap-to-donor site ratios of tissue perfusion indicating vascular flap compromise

**Figure 4:**
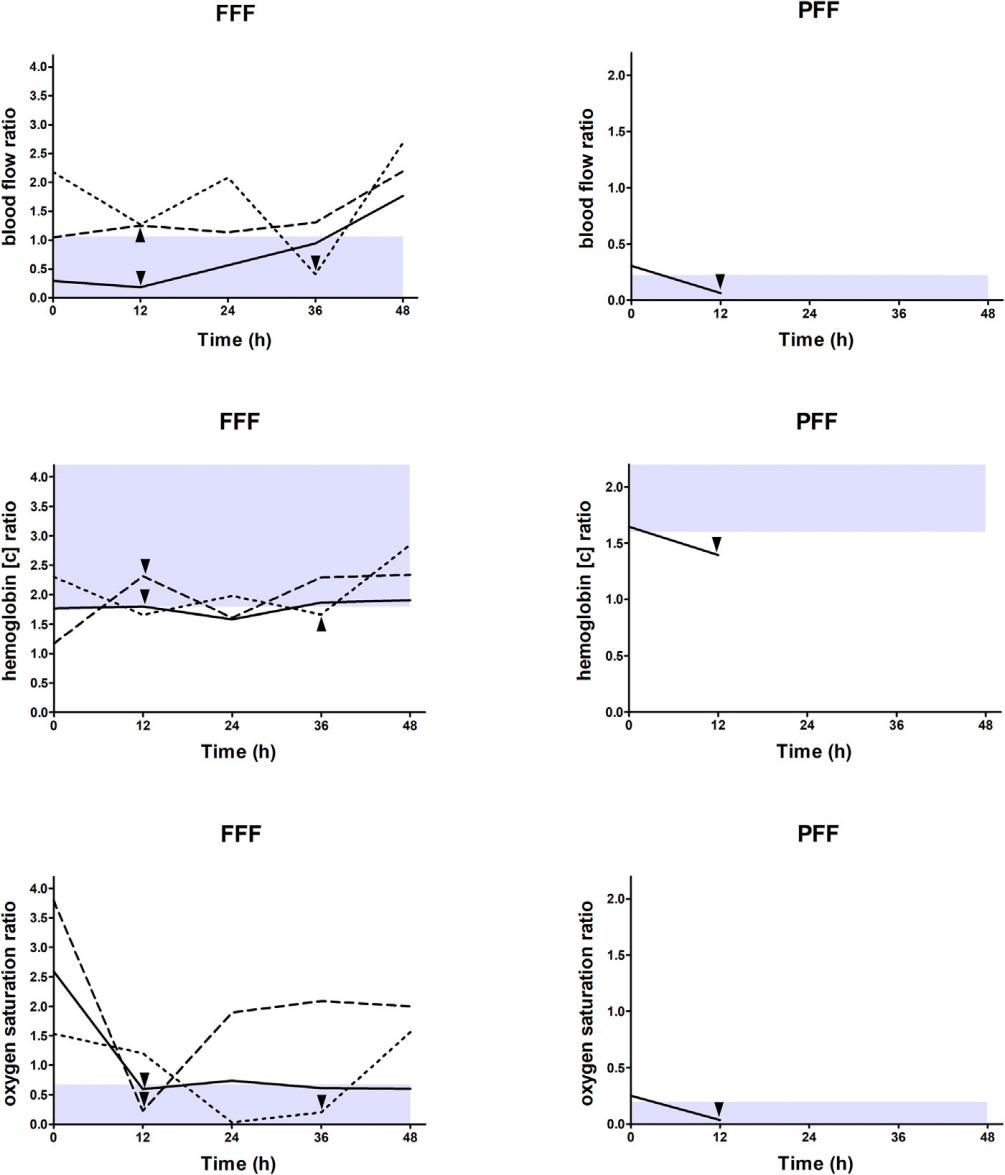
Data shown longitudinally for individual flaps as flap-to-donor site ratios for blood flow (top), hemoglobin concentration (middle), and hemoglobin oxygen saturation (bottom) for each measurement timepoint (0 h postoperatively, 12 h postoperatively, 24 h postoperatively, 36 h postoperatively, 48 h postoperatively) separately for three revised fasciocutaneous free flaps (FFF) (left) (revision 12 h, 12 h, and 36 h postoperatively) and one revised perforator free flaps (PFF) (right) (revision 12 h postoperatively); triangle = timepoint before flap revision, gray shaded area = values below (blood flow and hemoglobin oxygen saturation) or above (hemoglobin concentration) the cut-off values of the flap-to-donor site ratios of tissue perfusion indicating vascular flap compromise for blood flow (FFF and PFF: <1.063 and <0.224), hemoglobin concentration (FFF and PFF: >1.792 and >1.597) and hemoglobin oxygen saturation (FFF and PFF: <0.673 and <0.199) (determination based on the minimum (blood flow and hemoglobin oxygen saturation) or maximum (hemoglobin concentration) value of all 5 measurement timepoints); abbreviations: FFF, Fasciocutaneous free flap; PFF, Perforator free flap.

The cut-off values of the flap-to-donor site ratios of tissue perfusion indicating vascular flap compromise for blood flow, hemoglobin concentration and hemoglobin oxygen saturation for FFFs and PFFs were as follows: <1.063 and <0.224, >1.792 and >1.597, and <0.673 and <0.199, respectively (Table 4, Figure 4).

**Table 4:** Cut-off values indicating vascular flap compromise.

Cut-off value	AUC (CI)	Sensitivity (CI)	Specificity (CI)	PPV (CI)	NPV (CI)
FFF (*n* = 39)					
Blood flow flap-to-donor site ratio					
<1.063	0.880 (0.605–1.000)	1.000 (0.292–1.000)	0.667 (0.490–0.814)	0.200 (0.043–0.481)	1.000 (0.858–1.000)
Hemoglobin concentration flap-to-donor site ratio					
>1.792	0.907 (0.665–1.000)	1.000 (0.292–1.000)	0.722 (0.548–0.858)	0.231 (0.050–0.538)	1.000 (0.868–1.000)
Hemoglobin oxygen saturation flap-to-donor site ratio					
<0.673	1.000 (1.000–1.000)	1.000 (0.292–1.000)	1.000 (0.903–1.000)	1.000 (0.292–1.000)	1.000 (0.903–1.000)
PFF (*n* = 23)					
Blood flow flap-to-donor site ratio					
<0.224	1.000 (1.000–1.000)	1.000 (0.025–1.000)	1.000 (0.846–1.000)	1.000 (0.025–1.000)	1.000 (0.846–1.000)
Hemoglobin concentration flap-to-donor site ratio					
>1.597	0.864 (0.714–1.000)	1.000 (0.025–1.000)	0.864 (0.651–0.971)	0.250 (0.006–0.806)	1.000 (0.824–1.000)
Hemoglobin oxygen saturation flap-to-donor site ratio					
<0.199	1.000 (1.000–1.000)	1.000 (0.025–1.000)	1.000 (0.846–1.000)	1.000 (0.025–1.000)	1.000 (0.846–1.000)

Receiver operator characteristics analysis for blood flow flap-to-donor site ratio, hemoglobin concentration flap-to-donor site ratio, and hemoglobin oxygen saturation flap-to-donor site ratio for indicating vascular flap compromise based on data of revised flaps (*n* = 4) and non-revised flaps (*n* = 58) in terms of the minimum (blood flow and hemoglobin oxygen saturation) or maximum (hemoglobin concentration) value of all 5 measurement timepoints (all *p* < 0.001); theoretical optimal cut-off values were determined by calculation of the Youden Index (YI) (blood flow: FFF 0.667 and PFF 1.000; hemoglobin concentration: FFF 0.722 and PFF 0.864; hemoglobin oxygen saturation: FFF 1.000 and PFF 1.000); abbreviations: AUC, Area under the curve; CI, Confidence interval; PPV, Positive predictive value; NPV, Negative predictive value; FFF, Fasciocutaneous free flap; PFF, Perforator free flap.

## Discussion

For this study, the tissue perfusion of the contralateral donor site was chosen as the reference, as studies have shown similar tissue perfusion values for bilateral body sites.^27^^,^^28^ Attached surface probes were used for the O2C analysis system, as they offer the advantages of constant probe pressure and location during measurement and the potential to facilitate continuous postoperative flap monitoring due to their small size and light weight compared to surface probes measuring in 2- and 8-mm tissue depth.^29^, ^30^, ^31^ The surface probes were permanently attached with at the flap at a distance from the located perforator vessels in the PFFs to avoid damaging the perforator vessels. In this study, a time period of 48 h was chosen, as vascular flap compromise is likely to occur within this time interval.^32^

This study demonstrated that flap and contralateral donor site tissue perfusion generally differed, with higher blood flow, hemoglobin concentration, and hemoglobin oxygen saturation values at the flap.

The higher tissue perfusion values at the flap compared to the donor site are consistent with the results of a previous study that also used the O2C analysis system and showed that in FFFs and PFFs the perfusion of the flap tissue at the recipient site after microsurgical anastomosis was higher than the perfusion of the flap tissue at the donor site before flap harvesting.^33^ Furthermore, our study indicated that flap tissue perfusion was dynamic in the postoperative course, with increasing values for blood flow in FFFs and PFFs and decreasing values in FFFs or constant values in PFFs for hemoglobin concentration and hemoglobin oxygen saturation in non-revised flaps, which corresponds to the results of a previous study in which flap tissue perfusion was also measured postoperatively with the O2C analysis system.^14^ However, both earlier studies differed from the present study in two ways: they used measurement depths of 2 and 8 mm instead of 3 mm, with different measurement depths representing different tissue components with specific tissue perfusion characteristics, and the flap tissue perfusion was measured consecutively (at the donor site first followed by the recipient site) instead of simultaneously.^14^^,^^33^ The observed increased flap tissue perfusion at the recipient site after microsurgical anastomosis could generally be attributed to higher perfusion pressures in the cervical vasculature and lower vascular resistance in the denervated flap tissue at the recipient site.^14^^,^^15^^,^^19^^,^^34^

This study showed that the flap-to-donor site ratios of tissue perfusion were lower in terms of blood flow and hemoglobin oxygen saturation and higher in terms of hemoglobin concentration in revised flaps than in non-revised flaps.

These observations are consistent with the results of previous studies that used the O2C analysis system postoperatively with surface probes to measure tissue perfusion at 2- and 8-mm tissue depths for flap monitoring and showed that vascular flap compromise is associated with lower blood flow and hemoglobin oxygen saturation values, reflecting insufficient arterial inflow, and higher hemoglobin concentration values, reflecting insufficient venous outflow, both of which are critical for flap viability and thus flap survival.^5^, ^6^, ^7^^,^^14^ Similarly, the predefined thresholds indicating vascular flap compromise in postoperative flap monitoring with the O2C analysis system based on tissue perfusion measurement in 2- and 8-mm tissue depths were minimum blood flow and hemoglobin oxygen saturation values and maximum hemoglobin concentration values.^7^^,^^14^ Interestingly, regardless of the cause of the vascular flap compromise, the flap-to-donor site ratios of tissue perfusion were altered in all measured parameters in revised flaps compared to non-revised flaps, which could be due to a veno-arterial response, at least in the case of venous congestion, with a reduction in arterial inflow at a higher venous transmural pressure due to venous congestion.^35^

This study also demonstrated that flap-to-donor site ratios could be used to detect vascular flap compromise in postoperative flap monitoring. However, given the low number of flap revisions, the pooling of measurement timepoints for the receiver operator characteristics analyses, and the unstable prediction models in terms of the derived cut-off values, further studies are needed to confirm the determined cut-off values.

The determined cut-off values of flap-to-donor site ratios of tissue perfusion indicating vascular flap compromise corresponded to predefined thresholds established in previous studies using the O2C analysis system with flap tissue perfusion measurement at 2- and 8-mm tissue depths with absolute minimum blood flow and hemoglobin oxygen saturation values and relative maximum increases in hemoglobin concentration values.^7^^,^^14^ However, since this study used flap-to-donor site ratios of tissue perfusion and thus referred to donor site tissue perfusion as the reference, the threshold for hemoglobin concentration was also an absolute threshold.^7^^,^^14^ Apart from a potentially higher accuracy of the flap-to-donor site ratio in detecting vascular flap compromise, the discrepancy between the timing at which vascular flap compromise was indicated in this study, based on tissue perfusion measurement with the O2C analysis system at a 3-mm tissue depth in relation to flap-to-donor site ratio thresholds, and the timing at which flap revision was actually performed independently of this study, based on flap perfusion measurement with the O2C analysis system at 2- and 8-mm tissue depths in relation to predefined absolute or relative thresholds, may be due to an influence of the measurement depth on tissue perfusion values.^7^^,^^14^ Furthermore, it should be noted that reduced tissue perfusion at the contralateral donor site, e.g., due to smoking habits, may limit the use of flap-to-donor-site ratios for the detection of vascular flap compromise, as low tissue perfusion at the contralateral donor site would lead to a higher flap-to-donor-site ratio in terms of blood flow and hemoglobin oxygen saturation, and thus could potentially conceal lower tissue perfusion at the flap site due to vascular flap compromise.^9^^,^^27^

In general, it should be noted that additional measurement of donor site tissue perfusion is associated with additional effort during postoperative flap monitoring. However, the measurement is performed at the donor site in the same manner as at the flap site and does not require special training or changes to the device settings. In terms of clinical applicability, the surface probe could be fixed at the donor site with an adhesive tape during the complete monitoring interval, with the probe connected to the system during measurement.

The limitations of this study are the small number of patients with different flap types and the small number of revised flaps. However, the number of flaps requiring revision is generally low in microvascular head and neck reconstruction.^2^ In addition, although studies have shown similar tissue perfusion values between bilateral body sites, differences in tissue perfusion between bilateral donor sites cannot be ruled out, particularly regarding differences in the sensitivity of the vasculature to vasoactive catecholamines, complicating comparisons between the flap and donor sites when using the donor site as the reference.^19^^,^^27^, ^28^, ^29^, ^30^^,^^34^ Nevertheless, the placement of the surface probe on the flap, particularly in PFFs with a distance to the perforator vessel, might have influenced the flap perfusion measurement values. In general, the low number of vascular flap compromises due to arterial insufficiency, with only one case, the presence of each type of vascular flap compromise in only one flap type, i.e., venous congestion in FFFs and arterial insufficiency in PFFs, and the lack of prediction model validation, all limit the generalizability of the study results, particularly with regard to the defined thresholds, and point to the need for validation in future studies. In addition, data on variables that could potentially influence tissue perfusion and thus the study results, e.g., systemic blood pressure, vasoactive medication, temperature or sedation, were not available for the measurement timepoints.^15^^,^^20^

This exploratory study was conducted to evaluate the flap-to-donor site ratio of tissue perfusion with regard to the detection of vascular flap compromise in postoperative flap monitoring. In terms of clinical implications, it was found that the flap-to-donor site ratio of tissue perfusion could be used for the detection of vascular flap compromise by providing an orientation for absolute thresholds for blood flow, hemoglobin concentration, and hemoglobin oxygen saturation, potentially increasing the accuracy of flap monitoring based on the measurement of flap tissue perfusion. Nevertheless, given its exploratory nature, this study will not question the status of clinical examination as the gold standard for postoperative flap monitoring in microvascular head and neck reconstruction.^11^^,^^12^ Further studies are needed to confirm the thresholds that indicate vascular flap compromise.

## Conclusion

This study showed that the flap-to-donor site ratio of tissue perfusion could be used to detect postoperative vascular flap compromise in microvascular head and neck reconstruction. By providing absolute thresholds for blood flow, hemoglobin concentration, and hemoglobin oxygen saturation, the use of the flap-to-donor site ratio of tissue perfusion can potentially increase the accuracy of postoperative flap monitoring based on the measurement of tissue perfusion. However, further studies are needed to verify the determined thresholds and to demonstrate the potential benefit of increased accuracy in the light of the additional effort required to measure donor site tissue perfusion for postoperative flap monitoring.

## Funding

This research did not receive any specific grant from funding agencies in the public, commercial, or not-for-profit sectors.

## Ethical approval

This study was approved by the local ethics committee of the Medical Faculty of RWTH Aachen University (EK 22-358).

## Declaration of competing interest

The authors declare that they have no conflict of interest regarding the results published in this article.

## Appendix Supplementary materials

Image, application 1
